# Prevalence and genetic diversity of porcine circovirus type 2 in northern Guangdong Province during 2016–2021

**DOI:** 10.3389/fvets.2022.932612

**Published:** 2022-08-10

**Authors:** Wenjin Nan, Jingbo Wu, Honghui Hu, Guoliang Peng, Simin Tan, Zhibang Deng

**Affiliations:** ^1^Lab of Animal Disease Prevention & Control and Animal Model, Hunan Provincial Key Laboratory of Protein Engineering in Animal Vaccines, College of Veterinary Medicine, Hunan Agricultural University (HUNAU), Changsha, China; ^2^North Guangdong Collaborative Innovation and Development Center of Pig Farming and Disease Control, Shaoguan University, Shaoguan, China

**Keywords:** porcine circovirus type 2, epidemiology, complete genome, genetic characteristics, Guangdong Province

## Abstract

The emergence and widespread of porcine circovirus-associated diseases (PCVADs), mainly caused by porcine circovirus type 2 (PCV2), threatens the Chinese swine industry. In this study, to investigate the recent prevalence of PCV2 in northern Guangdong Province of China, 573 tissue samples from 132 pig farms were collected during 2016–2021 and analyzed *via* PCR. Overall, 51.38% (297/573, 95%CI 47.74–55.92) samples were tested PCV2 positive. The detection rate of PCV2 was significantly lower in samples collected before 2016-2018 than after the outbreak of African Swine Fever (2019-2021), being 59.85% (158/264, 95%CI 53.94–65.76) and 41.47% (141/340, 95%CI 36.43–46.71), respectively. On the other end, the genetic characteristics of 26 PCV2 strains were further analyzed. These PCV2 strains belonged to three genotypes, including PCV2a, PCV2b, and PCV2d. Specifically, the predominant genotype prevalent during two periods (2016–2018 and 2019–2021) wasPCV2b (81.82%, 9/11) and PCV2d (80.0%, 12/15), respectively. The results above illustrated the high prevalence and the genetic evolution feature of PCV2 in Guangdong Province in recent years.

## Introduction

Porcine circoviruses (PCVs) are small, circular, single-stranded DNA viruses belonging to the genus of *Circovirus* of the family *Circoviridae* (1). Currently, four genotypes of PCVs have been identified, termed porcine circovirus type 1 (PCV1), PCV2, PCV3, and PCV4 (2). Since the first identification of PCV2 in Canada in the 1990s, this pathogen has been considered the primary causative agent of porcine circovirus-associated diseases (PCVADs) (3, 4). PCVADs are mainly characterized by postweaning multisystemic wasting syndromes (PMWS), such as respiratory distress in piglets, slow growth in fattening pigs, reproductive failures in sows, etc., which threatens the development of pig industry worldwide (3, 4).

The PCV2 genome is 1,766–1,768 nucleotide (nt) in length, which mainly comprises two open reading frames (ORFs). The ORF1 encodes the replication-related proteins (Rep and Rep'), while the ORF2 encodes the capsid protein (Cap), which induces the neutralization antibodies production and viral entry (3, 5). According to the genomic characteristics of PCV2 strains, they are divided into eight subtypes (PCV2a-PCV2h) (6). PCV2 strains prevalent before 2008 belonged to three genotypes (PCV2a-PCV2c). The PCV2d was identified in 2010, and then prevalent in pig populations worldwide since 2012 (4). In recent years, other novel PCV2 genotypes (PCV2e-PCV2h) have been identified (5). Other than these, owing to the high sequence similarity among different PCV2 genotypes, the novel recombinant strains generated from different PCV2 genotype strains were also documented (7, 8).

A number of studies have investigated the epidemiology and genetic features of PCV2 in certain regions of China (9–11), which confirmed the rapid evolution of PCV2, and the genotype shift from PCV2b to PCV2d in China. The prevalence and genetic characteristics of PCV2 in Guangdong Province have been documented in previous studies (12, 13). However, the corresponding information for recent years is still missing, particularly after the outbreak of African Swine Fever (ASF). In this study, polymerase chain reaction (PCR) was performed to investigate the epidemiological characteristics of PCV2 in Guangdong Province from 2016 to 2021. Moreover, the complete genomes of 26 PCV2 strains from different periods were sequenced and analyzed.

## Materials and methods

### Sample collection

From April 2016 to September 2021, 573 tissue samples (lung, tonsil, and lymph node) were collected from 132 pig farms across the northern Guangdong Province (Shaoguan, Qingyuan, Heyuan, Zhaoqing, Guangzhou, and Meizhou cities). Most diseased pigs from these farms showed clinical symptoms characterized by PMWS and/or porcine dermatitis and nephropathy syndrome (PDNS) or reproductive failures. Tissue samples with detailed information including collection sites, collection dates, and clinical symptoms were sent to Shaoguan University for further processing.

### DNA extraction and PCV2 detection

Viral DNA genomes were extracted from the tissue sample using commercial kits (GDSBio, Guangdong, China) according to the manufacturer's instructions. Subsequently, the presence of PCV2 nucleic acid was detected by PCR with primers (PCV2-ORF2-P1 and PCV2-ORF2-P2) as described previously (14). The PCR products were analyzed in 1% agarose gel electrophoresis, in which the samples with the expected DNA bands, approximately 450 bp, were noted as PCV2-positive samples.

### PCV2 complete genome sequencing

According to the collected regions and years of the positive samples, 26 PCV2-positive samples were selected to amplify the complete genome of the virus with three pairs of primers (Table 1). Each PCR reaction (50 μl) contained 25 μl 2 × Taq Master mix (Takara Biotechnology, Dalian, China), 12.0 μl of each primer (10 pmol), 3.0 μl DNA template, and 20.0 μl sterilized water. The PCR amplification was performed with the following steps: 95°C for 5 min; 35 cycles of 95°C for 30 s, 55°C for 30 s, and 72°C for 60/90 s; followed by 72°C for 7 min. The PCR products were purified, cloned into the pUCm-T vector, and sequenced by Sangon Biotech Co. Ltd (Shanghai, China). The sequences of these novel PCV2 strains were submitted to the GenBank (ON361010-ON361035).

**Table 1:** Primers used in this study.

**Primer name**	**Sequence (5'−3')**	**Length**	**Annealing temperature**	**Purpose**
PCV2–P1–F:	TGTTTTCGAACGCAGTGCC	1045	55.0	Sequencing
PCV–P1–R	CCGTTGTCCCTGAGATCTAGGA			
PCV2–P2–F	GGACCCCAACCCCATAAAA	1254	55.0	Sequencing
PCV2–P2–R	CCCTCACCTATGACCCCTATGT			
PCV2–P3–F	GTACCTTGTTGGAGAGCGGG	1767~1678	55.0	Sequencing
PCV2–P3–R	TCACAGCAGTAGACAGGTCA			
PCV2–ORF2–P1	CACGGATATTGTAGTCCTGGT	449	52.0	Detection of PCV2 targeting to ORF2 gene
PCV2–ORF2–P2	CGCACCTTCGGATATACTGTG			

*If the complete genome of PCV2 was not successfully amplified using the primer PCV2–P3–F/R, two pairs of primers (PCV2–P1–F/R and PCV2–P2–F/R) were employed*.

### Bioinformatics analyses

The complete genomes of 20 reference PCV2 strains (including PCV2a, PCV2b, PCV2c, PCV2d, PCV2e, and PCV2h) were downloaded from the GenBank database (Supplementary Table S1). The nucleotide sequences and their corresponding amino acid sequences (Cap and Rep) variations of 26 novel PCV2 strains and reference strains were analyzed *via* the Lasergene DNAStar software. Phylogenetic trees were generated based on the complete genome and ORF2 gene sequences using the neighbor-joining method in the MEGA 7.0 software (Kimura 2-parameter model, 1,000 bootstrap replications).

### Data analyses

The statistical significance of the detection rates of PCV2 in pigs among different groups was analyzed using the chi-square test in the SPSS 21.0 software (SPSS Inc., Chicago, IL, USA), in which, *P*-values < 0.05 were taken as statistically significant. Meanwhile, the minimum infection rate (MIR) with 95% CI was determined *via* the SPSS 21.0 software.

## Results

### The epidemiology of PCV2 in northern Guangdong Province from 2016 to 2021

In this study, a total of 573 tissue samples from 132 pig farms were collected from northern Guangdong Province for the PCV2 nucleic acids test by conventional PCR. The results showed that 98 out of 132 (74.24%) investigated pig farms were tested PCV2-positive. Overall, 297 tissue samples (51.83%, 95%CI 47.74–55.92) were PCV2-positive, with the positive rates of PCV2 in different regions varying from 27.91–64.94% (Table 2). Moreover, The detection rate of PCV2 was significantly lower in samples collected before 2016–2018 than after the outbreak of African Swine Fever (2019–2021), being 59.85% (158/264, 95%CI 53.94–65.76) and 41.47% (141/340, 95%CI 36.43–46.71), respectively (Table 2). Additionally, the positive rate of PCV2 among pigs with PCVADs (58.45%, 95%CI 53.63–63.27) was significantly higher than in pigs without PCVADs (36.26%, 95%CI 29.05–43.47) (Table 2).

**Table 2:** Prevalence of PCV2 in pigs in northern Guangdong Province, China.

**Factor**	**Category**	**No. sample**	**No. positive**	**Prevalence (%) (95% CI)**	***P*–value**
Period	2014~2018	340	141	41.47 (36.43–46.71)	Reference
	2019~2021	264	158	59.85 (53.94–65.76)	<0.01
Symptom	PCVADs	402	235	58.45 (53.63–63.27)	<0.01
	Others	171	62	36.26 (29.05–43.47)	Reference
Region	Shaoguan	186	91	48.92 (41.74–56.10)	<0.01
	Qingyuan	96	60	62.50 (52.82–72.18)	<0.01
	Heyuan	119	58	48.74 (39.76–57.72)	<0.01
	Zhaoqing	77	50	64.94 (54.28–75.60)	<0.01
	Guangzhou	43	12	27.91 (14.50–41.32)	Reference
	Meizhou	52	26	50.0 (36.41–63.59)	<0.01
	Total	573	297	51.83 (47.74–55.92)	

### Genome sequence analysis

To further investigate the genetic features of recent PCV2 strains prevalent in Guangdong Province, 26 PCV2 strains were randomly selected among PCV2-positive samples from different regions, whose complete genome sequences were amplified, sequenced, and analyzed (Table 3). The complete genomes of all 26 novel PCV2 strains were 1,764–1,767 nt in length. Particularly, the length of the ORF1 gene encoding the Rep and ORF2 gene encoding the Cap was 945 nt and 702–705 nt, respectively. Pairwise-sequence comparisons among 26 novel isolates ranged from 95.9–99.9% (complete genome), 97.0–99.7% (ORF1), and 94.0–100.0% (ORF2) at the nt level, respectively, and 94.6–99.4% (Rep protein) and 98.6–100.0% (Cap protein) at amino acid (aa) level, respectively.

**Table 3:** Detail information of PCV2 strains obtained in this study, including strain name, collection year, isolation region, genotype, and GenBank accession numbers.

**Strain**	**Region**	**Collection year**	**Genotype**	**Accession number**
GD–HY−2016	Heyuan, Guangdong	2016	PCV2b	ON361010
GD–QY−2016	Qingyuan, Guangdong	2016	PCV2b	ON361011
GD–GZ−2016	Guangzhou, Guangdong	2016	PCV2b	ON361012
GD–ZQ−2016	Zhaoqing, Guangdong	2017	PCV2b	ON361013
GD–SG−2017	Shaoguan, Guangdong	2017	PCV2a	ON361014
GD–ZQ−2017	Zhaoqing, Guangdong	2017	PCV2d	ON361015
GD–QY−2017	Qingyuan, Guangdong	2017	PCV2b	ON361016
GD–HY−2018	Heyuan, Guangdong	2018	PCV2b	ON361017
GD–MZ−2018	Meizhou, Guangdong	2018	PCV2b	ON361018
GD–SG−2019	Shaoguan, Guangdong	2019	PCV2b	ON361019
GD–QY−2019	Qingyuan, Guangdong	2019	PCV2b	ON361020
GD–SG−2020–1	Shaoguan, Guangdong	2020	PCV2b	ON361021
GD–SG−2020–2	Shaoguan, Guangdong	2020	PCV2d	ON361022
GD–HY−2020	Heyuan, Guangdong	2020	PCV2d	ON361023
GD–MZ−2020	Meizhou, Guangdong	2020	PCV2d	ON361024
GD–ZQ−2020–1	Zhaoqing, Guangdong	2020	PCV2d	ON361025
GD–ZQ−2020–2	Zhaoqing, Guangdong	2020	PCV2d	ON361026
GD–GZ−2020	Guangzhou, Guangdong	2020	PCV2d	ON361027
GD–QY−2020	Qingyuan, Guangdong	2020	PCV2d	ON361028
GD–SG−2021–1	Shaoguan, Guangdong	2021	PCV2d	ON361029
GD–SG−2021–2	Shaoguan, Guangdong	2021	PCV2d	ON361030
GD–GZ−2021	Guangzhou, Guangdong	2021	PCV2d	ON361031
GD–HY−2021	Heyuan, Guangdong	2021	PCV2d	ON361032
GD–MZ−2021	Meizhou, Guangdong	2021	PCV2b	ON361033
GD–QY−2021	Qingyuan, Guangdong	2021	PCV2d	ON361034
GD–ZQ−2021	Zhaoqing, Guangdong	2021	PCV2b	ON361035

### Phylogenetic analysis

**Figure 1:**
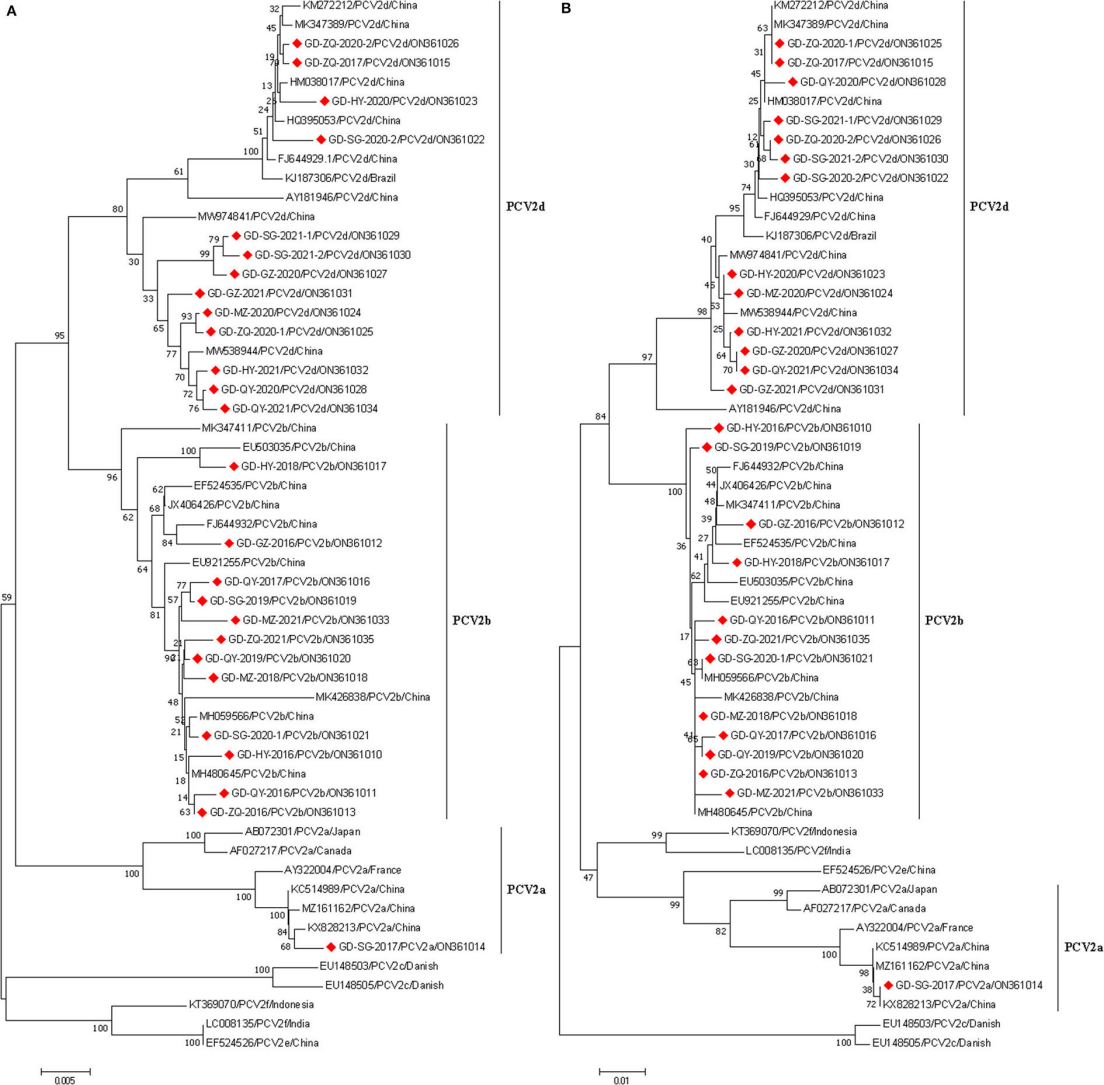
Phylogenetic analysis based on the complete genome **(A)** and ORF2 gene **(B)** sequences of 26 PCV2 strains obtained in this study and other reference strains. phylogenetic trees were constructed using the neighbor-joining method with 1,000 bootstrap replicates in MEGA7.0 software. Red squares represented PCV2 isolates obtained in this study.

Phylogenetic trees were generated according to the complete genome and ORF2 sequences of the 26 PCV2 strains in this study and 20 reference strains from GenBank. The results showed that the 26 Guangdong strains were divided into three sub-genotypes (PCV2a, PCV2b, and PCV2d) (Figures 1A,B), 12 of 26 (46.15%) isolates belonged to the genotype 2b; and half of all isolates belonged to the genotype 2d; only one isolate, GD-SG-2017, was clustered with PCV2a strains. Remarkably, the PCV2a isolate (GD-SG-2017) and 9 of 12 PCV2b isolates were collected between 2016 and 2018, whereas 92.31% (12/13) PCV2d strains were isolated during 2019-2021.

### Analysis of Cap amino acid sequences

To investigate the sequence characteristics of the Cap aa sequences obtained in this study, the aa sequence alignment was performed between the 26 PCV2 strains and the 20 reference PCV2 strains. The results showed that the lengths of Cap sequences of 26 PCV2 strains were 233 aa (12 PCV2b isolate and GD-GZ-2021) or 234 aa (12 PCV2d isolates and GD-SG-2017). As shown in Supplementary Table S2, a total of 8 and 4 aa substitutions were found in Cap among 12 PCV2b and 13 PCV2d isolates, respectively. To be more specific, these substitutions were located at sites 25 (R → H), 59 (K → R), 63 (R → K),84 (G → E), 131 (T → P), 151 (T → P), 173 (Y → H), and 190 (T → A) in the Cap of PCV2b isolates; and 30 (V → L), 169 (G → R), 206 (I → K), and 233 (N → K) in the Cap of PCV2d isolates, respectively. Remarkably, a series of unique aa substitutions in the Cap, for instance, the substitutions at sites 25, 59, and 63 were found in GD-GZ-2016 isolate; and site 151 in GD-QY-2016 isolate; and 59, 63, 84, 173, and 190 in GD-HY-2018 isolate, respectively.

## Discussion

PCV2 has been prevalent in pig populations in China for many years. The disease (PCVAD) caused by PCV2 is considered a major factor threatening the pig industry (11). Vaccine pressure, viral evolution, natural selection, and international pig transportation contribute to the rapid evolution rate of PCV2 (15). In recent years, two genotype shifts of PCV2 have been observed in China. PCV2a was the predominant prevalent genotype before 2003, which has been gradually replaced by PCV2b from 2003 to 2010. Owing to the high prevalence of PCV2 and the widely applied PCV2 vaccines since 2010, PCV2d is an emerging genotype prevalent in China (4, 16). Moreover, the co-prevalence of multiple PCV2 genotypes was often observed in the same region, even on the same pig farm (4, 7, 14, 17). Thus, an investigation of the prevalence and genetic characteristics of PCV2 will provide scientific evidence for the prevention and control of PCV2.

In this study, 573 samples from pigs were collected to investigate the epidemiological characteristics of PCV2 in the northern Guangdong Province of China. Several features were summarized: 1) high detection rate of PCV2 (51.83%, 297/573) was observed in nearly 75% of the investigated pig farms. The PCV2-positive rate among these samples in northern Guangdong Province was similar to other areas in China, such as Henan (62.4%, 73/117) (18), Shanghai (57.78%, 115/199) (4), and Yunnan (60.93%, 170/279) (11), but higher than that in Shandong Province (36.98%, 490/1325) (19); taken together, these bodies of evidence showed PCV2 high prevalence in China; 2) the positive rate of PCV2 among specimens collected before (2014–2018) was lower than that after (2019–2021) the outbreak of ASF. Owing to the prevalence of ASF in China (from 2019 to 2021), PCV2-positive pigs were introduced into pig farms to keep the breeding scale. Nevertheless, some pig farmers mainly focused on ASF prevention, but neglected PCV2 prevention, which resulted in the high prevalence of PCV2 from 2019 to 2021 in northern Guangdong province; 3) the PCV2 detection rate among pigs with PCVADs was higher than those without PCVADs. To better prevent the PCV2 spread, the PCV2 positive pigs without PCVADs should not be neglected.

Currently, PCV2 strains have been divided into eight genotypes (PCV2a–h) based on their genomic characteristics, in which, multiple genotypes were prevalent in China (4, 16). More recently, PCV2d has been considered a novel emerging major genotype prevalent in Chinese pig populations (15, 20). Among 26 PCV2 strains obtained in this study, only three genotypes were identified, PCV2a, PCV2b, and PCV2d, the proportions of which were 3.85% (1/26), 46.15% (12/26), and 50.0% (13/26), respectively, suggesting that PCV2b and PCV2d, rather than PCV2a, predominated in these investigated regions. According to the findings from other studies, more than 30% of PCV2 strains prevalent in China before 2018 belonged to the PCV2d genotype (16, 18), particularly, PCV2d has become the major dominant genotype circulating in Shandong Province from 2015 to 2018 (19). However, the results in this study showed that the predominant genotypes of PCV2 during 2014–2018 and 2019–2021 were PCV2b and PCV2d, the proportions of which were 81.82% (9/11) and 80.0% (12/15) in northern Guangdong Province, respectively. Guangdong Province is a major pig breeding area with millions of pigs transferred out, whereas very few pigs were introduced into this province before 2018. These factors resulted in the relatively stable PCV2b genotype in these areas. However, after the outbreak of ASF in China, a large number of pigs were introduced into Guangdong Province, which might lead to the PCV2 genotype shifting from PCV2b to PCV2d.

The PCV2 Cap is the sole structural protein that is responsible for a series of biological processes, such as virus entry into host cells, replication, and activation of host immune responses (21, 22). Therefore, the aa mutations in Cap may determine viral biological characteristics (23). In this study, a series of aa substitutions were found in the Cap protein (Supplementary Table S2), such as the sites of 30 (V → L) (*n* = 6), and 59 (K → R) (*n* = 2), and 63 (R → K) (*n* = 2). It has been confirmed that the aa residues 1–41 of Cap participate in viral nuclear localization (24), while the aa residues 47–63 are crucial for PCV2 epitope recognition (25). Further experiments will be performed to investigate the effects of these aa substitutions on PCV2 infection.

In conclusion, this study revealed the epidemiology and genetic characteristics of PCV2 in recent years in northern Guangdong Province of China and indicated the severe prevalence of PCV2 in these regions. Moreover, this study confirmed the prevalence of multiple PCV2 genotypes in Guangdong Province from 2016 to 2021, while, currently the PCV2d has become the predominant genotype. These findings highlight the importance to investigate the genetic features of currently prevalent PCV2 strains, and to develop novel vaccines to control PCV2d.

## Data availability statement

The datasets presented in this study can be found in online repositories. The names of the repository/repositories and accession number(s) can be found in the article/Supplementary material.

## Author contributions

Conceptualization: ZD. Methodology: WN, ST, JW, and HH. Software: WN and ST. Formal analysis and investigation: WN, ST, JW, GP, and HH. Resources and original draft writing: WN. Review and revised and funding acquisition: WN and ZD. All authors participated in the article editing and approved the final manuscript.

## Funding

This work was supported by Natural Science Foundation of Hunan Province (2021JJ30316) and the Project of Swine Innovation Team in Guangdong Modern Agricultural Research System (2021KJ126).

## Conflict of interest

The authors declare that the research was conducted in the absence of any commercial or financial relationships that could be construed as a potential conflict of interest.

## Publisher's note

All claims expressed in this article are solely those of the authors and do not necessarily represent those of their affiliated organizations, or those of the publisher, the editors and the reviewers. Any product that may be evaluated in this article, or claim that may be made by its manufacturer, is not guaranteed or endorsed by the publisher.
