# Supplementary material for: Prevalence and genetic diversity of porcine circovirus type 2 in northern Guangdong Province during 2016–2021
Source: Front Vet Sci. 2022 Aug 10;9:932612. doi: 10.3389/fvets.2022.932612 (PMC9399655; doi:10.3389/fvets.2022.932612)
Supplement: Supplementary file 1 [file Table_1.DOC]

| Accession number | Genotype | Region | Collection year |
| --- | --- | --- | --- |
| AB072301 | PCV2a | Japan | 2001 |
| AF027217 | PCV2a | Canada | 1998 |
| AY322004 | PCV2a | Franch | 2004 |
| KX828213 | PCV2a | South Korea | 2012 |
| KC514989 | PCV2a | China | 2012 |
| MZ161162 | PCV2a | China | 2021 |
| EF524535 | PCV2b | China | 2007 |
| EU503035 | PCV2b | China | 2008 |
| EU921255 | PCV2b | China | 2008 |
| FJ644932 | PCV2b | China | 2009 |
| JX406426 | PCV2b | China | 2011 |
| MK347411 | PCV2b | China | 2015 |
| MK426838 | PCV2b | China | 2018 |
| MH059566 | PCV2b | China | 2017 |
| MH480645 | PCV2b | China | 2017 |
| EU148503 | PCV2c | Denmark | 2007 |
| EU148505 | PCV2c | Denmark | 2007 |
| AY181946 | PCV2d | China | 2002 |
| FJ644929 | PCV2d | China | 2008 |
| HM038017 | PCV2d | China | 2008 |
| HQ395053 | PCV2d | China | 2010 |
| KJ187306 | PCV2d | Brazil | 2013 |
| KM272212 | PCV2d | China | 2014 |
| MK347389 | PCV2d | China | 2018 |
| MW538944 | PCV2d | China | 2019 |
| MW974841 | PCV2d | China | 2019 |
| EF524526 | PCV2e | China | 2007 |
| KT369070 | PCV2f | Indonesia | 2013 |
| LC008135 | PCV2f | India | 2012 |

**Supplementary Table 1**: The information of PCV2 reference strains used in this study.
