# Supplementary material for: Prevalence and genetic diversity of porcine circovirus type 2 in northern Guangdong Province during 2016–2021
Source: Front Vet Sci. 2022 Aug 10;9:932612. doi: 10.3389/fvets.2022.932612 (PMC9399655; doi:10.3389/fvets.2022.932612)
Supplement: Supplementary file 2 [file Table_2.DOC]

| Strain | Genotype | Amino acid sites | | | | | | | | | | | | | | | | | | |
| --- | --- | --- | --- | --- | --- | --- | --- | --- | --- | --- | --- | --- | --- | --- | --- | --- | --- | --- | --- | --- |
|  |  | 25 | 30 | 53 | 59 | 63 | 68 | 84 | 89-90 | 111 | 121 | 131 | 134 | 151 | 169 | 173 | 190 | 206 | 233 | 235 |
| GD-HY-2016 | PCV2b | R | V | F | K | R | A | G | RS | T | S | T | T | T | S | Y | T | I | N | - |
| GD-QY-2016 | PCV2b | R | V | F | K | R | A | G | RS | T | S | T | T | P | S | Y | T | I | N | - |
| GD-GZ-2016 | PCV2b | H | V | F | R | K | A | G | RS | T | S | P | T | T | S | Y | T | I | N | - |
| GD-ZQ-2016 | PCV2b | R | V | F | K | R | A | G | RS | T | S | T | T | T | S | Y | T | I | N | - |
| GD-QY-2017 | PCV2b | R | V | F | K | R | A | G | RS | T | S | T | T | T | S | Y | T | I | N | - |
| GD-SG-2017 | PCV2a | R | V | I | K | R | N | G | LT | T | T | T | N | T | R | Y | T | I | N | K |
| GD-HY-2018 | PCV2b | R | V | F | R | K | A | E | RS | T | S | T | T | T | S | H | A | I | N | - |
| GD-MZ-2018 | PCV2b | R | V | F | K | R | A | G | RS | T | S | T | T | T | S | Y | T | I | N | - |
| GD-SG-2019 | PCV2b | R | V | F | K | R | A | G | RS | T | S | T | T | T | S | Y | T | I | N | - |
| GD-QY-2019 | PCV2b | R | V | F | K | R | A | G | RS | T | S | T | T | T | S | Y | T | I | N | - |
| GD-SG-2020-1 | PCV2b | R | V | F | K | R | A | G | RS | T | S | T | T | T | S | Y | T | I | N | - |
| GD-ZQ-2021 | PCV2b | R | V | F | K | R | A | G | RS | T | S | T | T | T | S | Y | T | I | N | - |
| GD-MZ-2021 | PCV2b | R | V | F | K | R | A | G | RS | A | S | T | T | T | S | Y | T | I | N | - |
| GD-ZQ-2017 | PCV2d | R | V | I | K | R | N | G | LT | T | T | T | N | T | S | Y | T | I | N | K |
| GD-SG-2020-2 | PCV2d | R | V | I | K | R | N | G | LT | T | T | T | N | T | R | Y | T | I | N | K |
| GD-HY-2020 | PCV2d | R | L | I | K | R | N | G | LT | T | T | T | N | T | G | Y | T | I | N | K |
| GD-MZ-2020 | PCV2d | R | L | I | K | R | N | G | LT | T | T | T | N | T | G | Y | T | I | N | K |
| GD-ZQ-2020-1 | PCV2d | R | V | I | K | R | N | G | LT | T | T | T | N | T | R | Y | T | I | N | K |
| GD-ZQ-2020-2 | PCV2d | R | V | I | K | R | N | G | LT | T | T | T | N | T | G | Y | T | I | N | K |
| GD-GZ-2020 | PCV2d | R | L | I | K | R | N | G | LT | T | T | T | N | T | G | Y | T | I | N | K |
| GD-QY-2020 | PCV2d | R | V | I | K | R | N | G | LT | T | T | T | N | T | G | Y | T | I | N | K |
| GD-SG-2021-1 | PCV2d | R | V | I | K | R | N | G | LT | T | T | T | N | T | G | Y | T | I | N | K |
| GD-SG-2021-2 | PCV2d | R | V | I | K | R | N | G | LT | T | T | T | N | T | G | Y | T | I | N | K |
| GD-GZ-2021 | PCV2d | R | L | I | K | R | N | G | LT | T | T | T | N | T | G | Y | T | K | K | - |
| GD-HY-2021 | PCV2d | R | L | I | K | R | N | G | LT | T | T | T | N | T | G | T | I | I | N | K |
| GD-QY-2021 | PCV2d | R | L | I | K | R | N | G | LT | T | T | T | N | T | G | T | I | I | N | K |

**Supplemental Table 2:** Comparison of Cap amino acid sequences of PCV2 strains identified in the present study.
